# Tempol Protects Against Acute Renal Injury by Regulating PI3K/Akt/mTOR and GSK3β Signaling Cascades and Afferent Arteriolar Activity

**DOI:** 10.1159/000490338

**Published:** 2018-05-30

**Authors:** Gensheng Zhang, Qin Wang, Wenwen Wang, Minghua Yu, Suping Zhang, Nan Xu, Suhan Zhou, Xiaoyun Cao, Xiaodong Fu, Zufu Ma, Ruisheng Liu, Jianhua Mao, En Yin Lai

**Affiliations:** a Department of Physiology, and the Children’s Hospital, Zhejiang University School of Medicine, Hangzhou; b Department of Pathology, Women’s Hospital, Zhejiang University School of Medicine, Hangzhou; c Department of Physiology, School of Basic Medical Sciences, Guangzhou Medical University, Guangzhou; d Department of Nephrology, Tongji Hospital of Tongji Medical College, Huazhong University of Science and Technology, Wuhan, China; e Department of Molecular Pharmacology & Physiology, University of South Florida College of Medicine, Tampa, Florida, USA

**Keywords:** Acute kidney injury, Ischemia/reperfusion, Tempol, Afferent arteriole

## Abstract

**Background/Aims:**

Free radical scavenger tempol is a protective antioxidant against ischemic injury. Tubular epithelial apoptosis is one of the main changes in the renal ischemia/reperfusion (I/R) injury. Meanwhile some proteins related with apoptosis and inflammation are also involved in renal I/R injury. We tested the hypothesis that tempol protects against renal I/R injury by activating protein kinase B/mammalian target of rapamycin (PKB, Akt/mTOR) and glycogen synthase kinase 3β (GSK3β) pathways as well as the coordinating apoptosis and inflammation related proteins.

**Methods:**

The right renal pedicle of C57Bl/6 mouse was clamped for 30 minutes and the left kidney was removed in the study. The renal injury was assessed with serum parameters by an automatic chemistry analyzer. Renal expressions of Akt/mTOR and GSK3β pathways were measured by western blot in I/R mice treated with saline or tempol (50mg/kg) and compared with sham-operated mice.

**Results:**

The levels of blood urea nitrogen (BUN), creatinine and superoxide anion (O_2_^.-^) increased, and superoxide dismutase (SOD) and catalase (CAT) decreased significantly after renal I/R injury. However, tempol treatment prevented the changes. Besides, I/R injury reduced renal expression of p-Akt, p-GSK3β, p-mTOR, Bcl2 and increased NF-κB, p-JNK and p53 in kidney, tempol significantly normalized these changes. In addition, renal I/R injury reduced the response of afferent arteriole to Angiotensin II (Ang II), while tempol treatment improved the activity of afferent arteriole.

**Conclusion:**

Tempol attenuates renal I/R injury. The protective mechanisms seem to relate with activation of PI3K/Akt/mTOR and GSK3β pathways, inhibition of cellular damage markers and inflammation factors, as well as improvement of afferent arteriolar activity.

## Introduction

Acute kidney injury (AKI) is a common and severe clinical disease. AKI occurs in approximately 5% of hospitalized patients and the overall mortality of AKI is estimated to range between 45–70%[1–3]. AKI is responsible for approximately 2 million deaths annually worldwide [2, 4, 5]. Renal ischemia/reperfusion (I/R) injury is one of the major causes of AKI in clinics which could be happen in patients with kidney transplantation, crossclamping surgery, embolism in renal arteries and shock [6–8]. Although AKI has been studied extensively, the molecular mechanisms involved in renal injury are not fully addressed. Intracellular reactive oxygen species (ROS], pro-inflammatory cytokines and pro-apoptotic elements have been demonstrated to play an essential role in the development of AKI[9, 10].

The phosphoinositide 3-kinase/protein kinase B (PI3K/PKB, Akt) pathway has been shown to play a critical role in regulating mitogenic signaling, apoptosis, cell proliferation and survival in different systems [11, 12]. PI3K/Akt pathway mediates cell survival in renal cells [13], in which mammalian target of rapamycin (mTOR) and glycogen synthase kinase 3β(GSK-3β) have been reported to play key role in Akt-mediated cell survival. The mTOR is an ubiquitously expressed intracellular serine/threonine protein kinase that plays a crucial role in regulating cell proliferation and organ growth by affecting many cellular processes [14]. GSK-3β is a serine/threonine kinase that participates in the regulation of cellular function [15]. GSK3β controls several extra-metabolic processes that are impaired in AKI and diabetes, including cytoskeletal dynamics, gene expression, proliferation, and apoptosis [16–18].

Oxidative stress caused by increased production of ROS leads to necrosis, apoptosis, inflammation and other disorders in AKI [19]. ROS could cause damage of renal tubular epithelial cells [20]. The redox-cycling antioxidant tempol can act as a superoxide dismutase (SOD) and a catalase member [21], thereby reducing tissue levels of both superoxide (O_2_^-^) and hydrogen peroxide (H_2_O_2_). It has been demonstrated to reduce the renal dysfunction caused by I/R injury, mainly through its free radical scavenging activity [22, 23] and improve the post-ischemic renal injury by inhibiting the neural activity of renal sympathetic nerve and ET-1 overproduction [24]. However, the detailed mechanism has not been fully elucidated. Thus, the aim of the present investigation is to study the detail protective mechanisms of tempol in renal I/R injury and its interaction with PI3K/Akt/mTOR and GSK3β pathways.

## Materials and Methods

### Reagents

Unless otherwise stated, all reagents were obtained from Sigma-Aldrich (St Louis, MO, USA).

### Animals

Male C57Bl/6 mice (25–28 g, SLAC laboratory animal company, Shanghai, China) were housed under climate-controlled condition with a 12 h light/dark cycle and provided with standard diet and water. An acclimation period of at least 1 week was provided before initiating experimental protocols. All protocols and animal handling procedures were performed in accordance with the National Institutes of Health (NIH, USA) guidelines for the care and use of laboratory animals and were approved by the Institute Animal Care and Ethical Committee of Zhejiang University School of Medicine.

### Renal ischemia/reperfusion model

Tempol (50 mg/kg, intraperitoneal injection) [25] was administered at 1 hour prior to the renal I/R injury. Mice were randomly divided into three groups (n=8 per group): sham, I/R and tempol+I/R group. Each mouse was anaesthetized with inhaled isoflurane. The right renal pedicle was clamped for 30 minutes and left nephrectomy performed to induce renal injury. After ischemia, the clamp was released for reperfusion. After the abdominal wounds being sutured, the mice were kept on a heating pad until its full consciousness. Sham group was dissected as above without clamping of the renal pedicle and left nephrectomy. The mouse temperature was monitored and maintained at 37 ± 0.5°C throughout the experiment with a heating pad. Mice were decapitated at 24 h after reperfusion, and the kidneys were harvested for further analysis.

### Measurement of renal injury markers

Blood was collected from the inferior vena cava and centrifuged at 3000 rpm, 4°C for 15 min. Serum creatinine and blood urea nitrogen (BUN) were tested by an automatic biochemical analyzer. The tissue concentrations of superoxide dismutase (SOD), catalase (CAT) and O_2_’ were measured by using standard assays according to manufacture instructions (SOD and CAT Assay Kit were purchased from Beyotime Biotechnology and O_2_’ Assay Kit from Najing Jiancheng Bioengineering Institute, Nanjing, China).

### Histological study

The kidney samples were harvested and fixed in 4% paraformaldehyde solution 24 h after renal I/R injury. Fixed kidney tissues were embedded in paraffin and were sliced and stained with Periodic Acid Schiff (PAS). Ten randomly chosen fields in cortex were captured under ×200 magnification. The percentage of necrotic tubules in each image was quantified as reported [8, 26, 27]. All morphometric analyses were performed in a blinded manner.

### Protein extraction and Western blotting

After euthanatized, the kidneys were collected and stored at −80°C. Frozen kidney tissue samples were homogenized in buffer containing 50 mM Tris-HCl, 0.5% Triton, 4mM EGTA, 10mM EDTA, 1mM Na_3_VO_4_, 30mM sodium pyrophosphate, 50 mM NaF, 1 mM phenylmethylsulfonyl fluoride, 50 μg/ml leupeptin, 25 μ/ml pepstatin A, 50μg/ml trypsin inhibitor and 1mmol/L dithiothreitol. The homogenate was centrifuged 10min at 13, 000g at 4°C. After determining supernatant protein concentration using Bradford’s solution, samples were boiled for 3 min in Laemmli’s sample buffer as described previously [28–30]. Samples containing equivalent amounts of protein were analyzed by 10% sodium dodecyl sulfate-polyacrylamide gel electrophoresis as described previously [31, 32]. Proteins were transferred to the immobile PVDF transfer membrane for 1h at 50V. Membranes were blocked in 20 mM Tris-HCl, 150mM NaCl and 0.1% Tween 20 containing 5% fat-free milk powder at room temperature for 1 h and immunodetected with antibodies to β-actin, p53, Bcl2, NF-κB, mTOR, p-mTOR (Abcam Cambridge), Akt, p-Akt (Ser473), GSK3β, p-GSK3β (Ser9), JNK and p-JNK (Cell Signaling Technology). Membranes were incubated with the appropriate horseradish peroxidase conjugated secondary antibody (1:5000). Immunoreactivity was incubated by enhanced chemiluminescence and visualized in an automated imaging analysis system (Tanon 5200 Multi, Tanon Science & Technology, Shanghai).

### Isolation and microperfusion of afferent arterioles

**Fig. 1.**
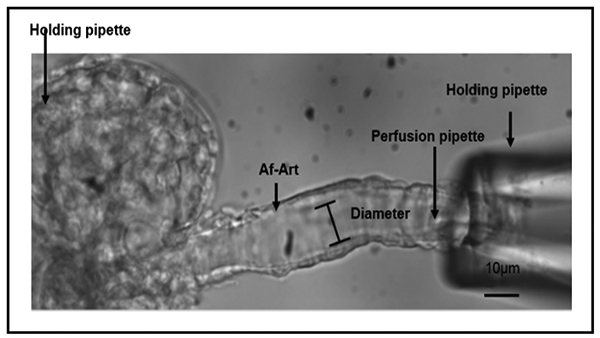
The renal afferent arteriolar microperfusion.

The isolation and perfusion of the afferent arterioles were similar as described previously [33, 34]. Briefly, mice were anesthetized with inhaled isoflurane and kidneys were removed and sliced quickly. Kidney slice was placed in ice-cold DMEM. Glomerulus with afferent arteriole was dissected under a microscope and transferred to a temperature-regulated chamber mounted on an inverted microscope (SZX16, Olympus, Japan) with DMEM (Fig. 1). The glomerulus was held with micropipette and afferent arteriole was cannulated and perfused with a set of micropipettes. The intraluminal pressure of the perfused afferent arteriole was maintained at 60 mmHg during experiment.

### Statistical analysis

The significance between the different groups was determined using a one-way ANOVA and accepted as statistically significant at *p*<0.05. All the results were presented as mean ± SEM.

## Results

### Effects of tempol on renal function after I/R injury

**Fig. 2.**
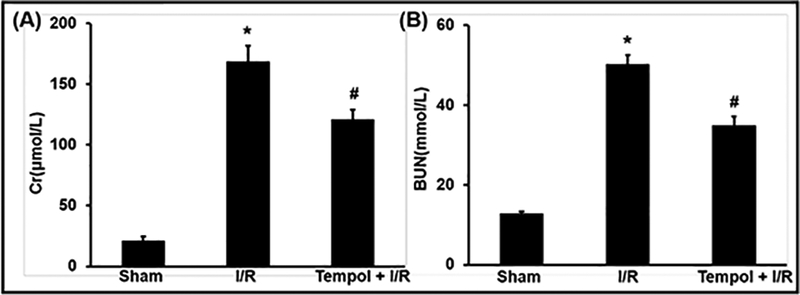
Effects of tempol on renal function after renal I/R injury. (A) Effects of tempol on serum creatinine after renal I/R injury. (B) Effects of tempol on serum BUN after renal I/R injury. Data are expressed as mean ± SEM, (n=8), * p<0.05 vs sham group; # p<0.05 vs I/R group.

The serum creatinine and BUN increased after renal I/R injury in mice and reduced by tempol (Fig. 2).

### Effect of tempol on oxidative stress related indicator levels after I/R injury

**Fig. 3.**
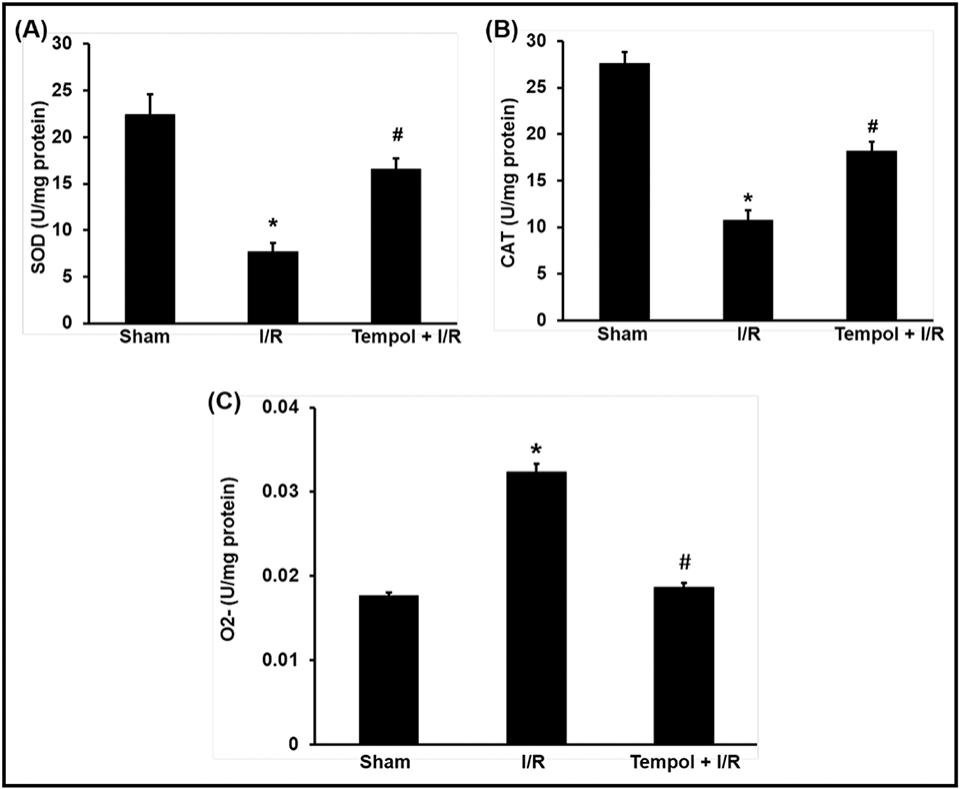
Effects of tempol on oxidative stress induced by renal I/R injury. SOD, CAT and O_2_^-^ in kidney were expressed as mean ± SEM, (n=8). * p<0.05 vs sham group; # p<0.05 vs I/R group.

SOD is an oxidoreductase that catalyzes the reaction between superoxide anions and hydrogen to yield molecular oxygen and hydrogen peroxide. Large numbers of oxygen free radicals are generated during periods of ischemia followed by reperfusion, leading to excessive SOD consumption [35]. CAT catalyzes the decomposition of hydrogen peroxide to water and oxygen [36]. They are very important enzymes in protecting the cell from oxidative damage by ROS. Surprisingly, I/R injury resulted in a significant reduction of SOD and CAT activity in kidney tissues and a significant increase of O_2_^-^, while tempol prevented the decrease of SOD and CAT activity and the increase of O_2_^-^(Fig. 3). These results suggested that tempol represses ROS accumulation by enhancing SOD and CAT activity and eliminate O_2_^-^ level.

### Effects of tempol on renal morphology after I/R injury

**Fig. 4.**
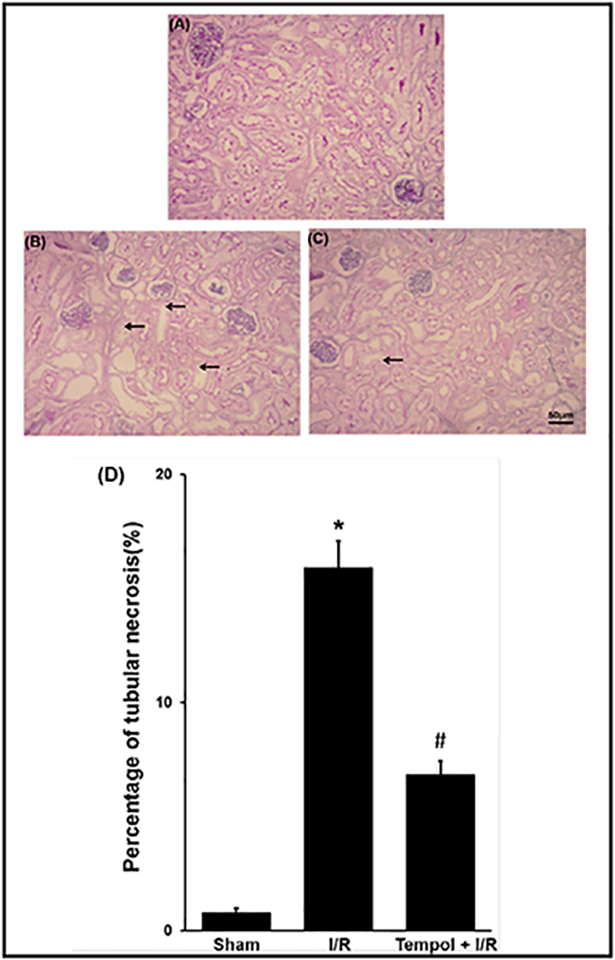
Effect of tempol on the morphologic changes of kidney tissues. PAS staining kidney sections were taken from sham group (A), I/R group (B) and I/R pretreated with tempol (50 mg/kg, C) and kidney injury was quantitatively measured by percentage of tubular necrosis in the cortex (D). Data were expressed as mean ± SEM, n=3, * p<0.05 vs sham group, # p <0.05 vs I/R group.

PAS staining of kidney sections in I/R group exhibited wide spread necrosis of tubular epithelial cells, with a large number of tubular and necrotic cells found inside the tubular cavity. The tempol pretreatment group only showed focal tubular necrosis and edema significantly reduced. The control group showed normal renal tissue structures (Fig. 4 A, B, C and D).

### Effect of tempol on the expression of PI3K/Akt/mTOR and GSK3βpathways after I/R injury

**Fig. 5.**
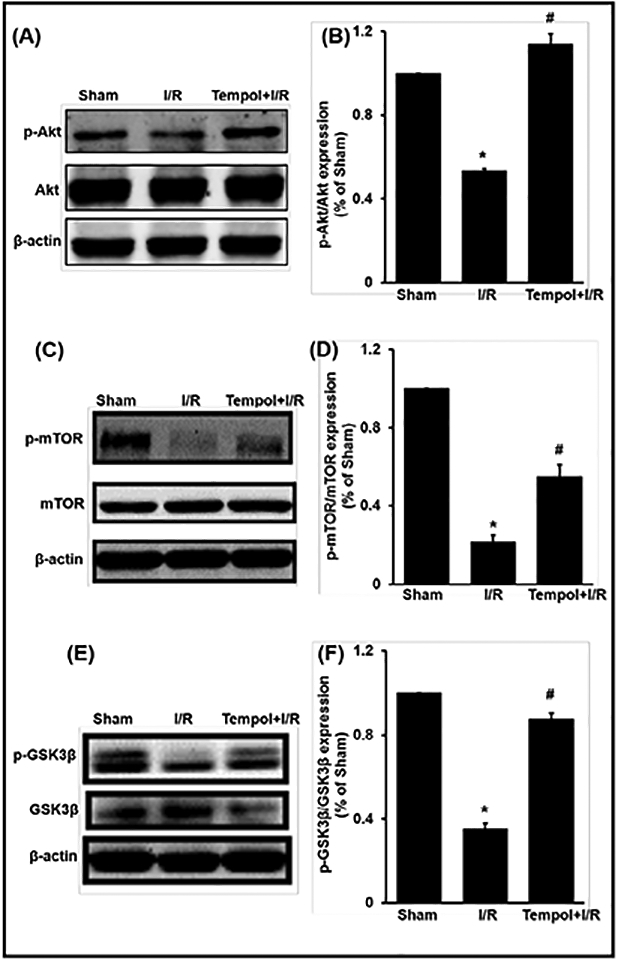
Effect of tempol on the expression of Akt/mTOR and GSK3β pathways in I/R injury. Data were expressed as mean ± SEM, n=5, * p <0.05 vs sham group; # p <0.05 vs I/R group.

Renal I/R injury reduced p-Akt (Fig. 5 A and B], p-mTOR (Fig. 5 C and D] and p-GSK3β expression (Fig. 5 E and F). Pretreatment with tempol reversed the reduction of p-Akt, p-mTOR and p-GSK3β expression.

### Effect of tempol on the expression of apoptosis and inflammation related proteins after I/R injury

JNK/phosphorylated-JNK (JNK/p-JNK) regulates several important cellular functions including cell growth, differentiation, survival and apoptosis [37]. Bcl-2 is a kind of inhibitor of the cell apoptosis and could prevent the apoptosis induced by free radicals and lipid peroxidation [38, 39]. Bcl-2 has the antioxidative characteristics in cells through participating the reduction action and inhibiting the formation of ROS [40]. The p53 is a tumor suppressor gene that plays an important role in cell cycle control and apoptosis. When tissue is exposed to I/R injury, the p53 activates the apoptotic pathway and directs cell death via apoptosis in order to protect the genome [41]. NF-κB is a protein complex that controls transcription of DNA, cytokine production and cell survival. NF-κB is recognized to have a potential role in apoptosis and the adaptive response to stress [42, 43].

**Fig. 6.**
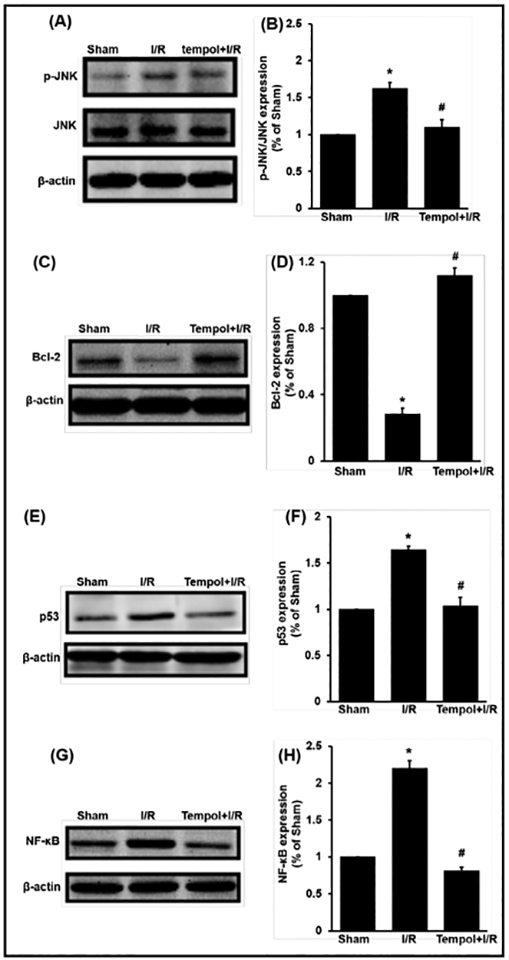
Effect of tempol on the expression of apoptosis and inflammation pathways in renal I/R injury. Data were expressed as mean ± SEM, n=5, * p <0.05 vs sham group; # p <0.05 vs I/R group.

Our data showed that the expression of Bcl2 was reduced, but p-JNK, p53 and NF-κB were increased after renal I/R injury. These changes were reduced in mice pretreated with tempol (Fig. 6).

### Effect of tempol on afferent arteriolar contraction to Ang II in different groups

**Fig. 7.**
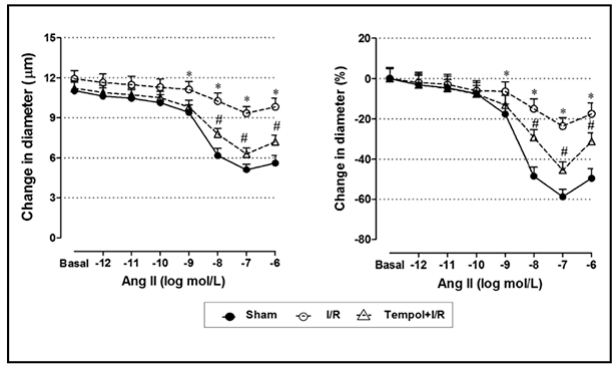
Effect of tempol on the afferent arteriolar activity in renal I/R injury. A) The representative picture of microperfusion. Dose response curve for Ang II in three different groups. Arteriolar luminal diameters were given in μm (B) and contractive percent of the control diameter (C). Data were expressed as mean ± SEM, n=5, * p <0.05 vs sham group; # p <0.05 vs I/R group.

After 30 min equilibration period, cumulative dose response curves of Ang II (10^−12^ to 10^−6^ mol/L) were obtained. Each concentration of Ang II was perfused for 2 minutes and recorded the constrictive response. In the sham group, Ang II at concentration of 10^−9^ mol/L and above induced dose-dependent contraction of afferent arteriole. However, this was reduced significantly in the afferent arteriole after I/R injury. Pretreatment with tempol significantly reversed the reduction of afferent arteriolar contraction (Fig. 7).

## Discussion

The important findings of the present study include: (1) I/R renal injury decreased SOD and CAT enzymatic activities and tempol improved these changes. (2) tempol corrected the expression of the apoptosis and inflammation pathway related proteins of Bcl-2, p53 p-JNK/JNK and NF-κB. (3) tempol restored p-mTOR, p-GSK3β and p-Akt activation. (4) tempol improved the afferent arteriolar contraction to Ang II.

It is well-known that NO participates in the pathophysiology of AKI and plays a great role in renal I/R injury. Imbalance between O_2_^-^ and NO is an important pathogenesis of I/R-induced AKI [44]. Tempol is reported to attenuate renal dysfunction by protecting tubular cells from oxidative stress [22, 23, 25, 45]. Indeed, tempol could improve tissue NO and PO_2_, reduce inflammation and protect the kidney from I/R-induced renal injury [22, 45]. Pretreatment with tempol may reduce the renal injury by inhibiting the neural activity of renal sympathetic nerve [24]. Our results confirm the reports that tempol protects the kidney from ischemic damage, but shows a complex mechanism.

The present study demonstrated an important mechanism of cellular defense after tempol involved up-regulation of Akt and its downstream genes, including mTOR and GSK3β. Tempol may restored Akt-dependent cytoprotective pathways by reducing the levels of ROS, lipid peroxidation [25, 46], inflammation and apoptosis [47], as well as upregulating SOD and CAT activity. Upregulating Bcl-2 expression in the tempol-treated mice after renal ischemia reduces kidney I/R injury by suppression of mitochondrial apoptosis [48, 49].

The microperfused afferent arteriolar technique *in vitro* has several advantages over other methodologies used to study the vessels in kidney. Renal afferent arterioles are the primary site of resistance in the renal circulation, their response to Ang II is very important to kidney function. Afferent arteriolar activity is a result of a number of factors. In the present study, we found that the Ang II responses were reduced in afferent arterioles in mice after I/R injury. This is in agreement with previous reports that renal microvascular reactivity reduced after the acute kidney injury [33, 50, 51].

## Conclusion

Our findings using the renal I/R injury model demonstrated that tempol effectively prevented against I/R-induced renal injury through PI3K/Akt/mTOR and GSK3β signaling pathways and improved the afferent arteriolar activity in mice.
